# Prediction of quality of life in schizophrenia using machine learning models on data from clinical antipsychotic trials of intervention effectiveness (CATIE) schizophrenia trial

**DOI:** 10.1192/j.eurpsy.2021.423

**Published:** 2021-08-13

**Authors:** M. Beaudoin, S. Potvin, A. Hudon, C.-E. Giguère, A. Dumais

**Affiliations:** 1 Psychiatry And Addictology, University of Montreal, Montreal, Canada; 2 Centre De Recherche, Institut universitaire en santé mentale de Montréal, Montréal, Canada; 3 Faculty Of Medicine, McGill University, Montreal, Canada; 4 Psychiatry, Institut national de psychiatrie légale Philippe-Pinel, Montreal, Canada

**Keywords:** schizophrénia, quality of life, machine learning, predictive models

## Abstract

**Introduction:**

Schizophrenia is a chronic and severe mental disorder. While research focus remains mainly on negative outcomes, it is questionable whether we are placing enough emphasis on improving their sense of well-being and functioning. This could be accessed through the study of the quality of life (QoL). To date, QoL prediction models mainly focused on neurocognition and psychotic symptoms, but their predictive power remained limited.

**Objectives:**

The aim is to accurately predict the QoL within schizophrenia using unsupervised learning methods.

**Methods:**

We computed variables from 952 patients from the CATIE study, a randomized, double-blind clinical trial for schizophrenia treatment. QoL was measured using the Heinrichs-Carpenter Quality of Life Scale and potential predictors included almost all available variables: symptoms, neurocognition, medication adherence, insight, adverse effects, etc. By optimizing parameters to reach optimal models, three linear regressions were calculated: (1) baseline predictors of 12-month QoL, (2) 6-month predictors of 12-month QoL, and (3) baseline predictors of 6-month QoL. Adjustments were made to ensure that included variables were not collinear nor redundant with QoL.

**Results:**

Calculated models had adjusted R-squared of 0.918, 0.922 and 0.913, respectively. Best predictors were medication side effects, sociodemographic and neurocognitive variables. Low psychotic and depressive symptoms were also included, as well as lab values suggesting the absence of problems with chloremia and calcemia.

**Conclusions:**

Calculated predictive models explain almost all subsequent QoL. It appears that physical health variables, generally omitted from mental health-related studies, have an important impact on patients’ QoL. Therefore, interventions should also consider these aspects.

**Disclosure:**

No significant relationships.

